# Call for papers: Generating stronger evidence to inform policy and practice: natural experiments on built environments, health behaviours and chronic diseases

**DOI:** 10.24095/hpcdp.44.5.06

**Published:** 2024-05

**Guest editors: **Dr. Stephanie Prince Ware (Public Health Agency of Canada), Dr. Gavin McCormack (University of Calgary)

**HPCDP Journal Editors:** Robert Geneau and Margaret de Groh (Public Health Agency of Canada)

Where we work, learn, play, eat and live has important implications for health. The built environment has been associated with the development of chronic disease, and with health behaviours often seen as critical pathways for this relationship.^1^,^2^ Built environments refer to components of the physical environment that are human-made or human-modified and include structures and buildings, recreation facilities, green spaces and parks, transportation systems and community design. 

Natural experiments are interventions that occur without a researcher’s ability to manipulate the intervention or exposure to the intervention.^3^,^4^ Natural experiments offer the opportunity to evaluate the effects of “naturally occurring” interventions such as changes to the built environment (e.g. creation of a new bike path, park improvements, infrastructure changes to schools or workplaces, construction of a new recreation facility or grocery store) on health behaviours and chronic disease risk. Natural experiments are often more practical for investigating the health impacts of environmental interventions when compared to traditional experimental studies (e.g. randomized controlled trials). Compared to cross-sectional studies, natural experiments provide a means to generate rigorous evidence to better establish causality, as well as to understand the implementation of interventions in “real-world” scenarios. 

This special issue answers the 2017 Canadian Public Health Officer annual report’s call to further evaluate the health impacts of community design features in Canada.^5^ This special issue resonates with the expanding scholarly and policy-oriented interest in the utility of natural experiments as a critical tool in advancing the body of evidence and for informing interventions to improve public and population health.^6^,^7^ Specifically, the objective of this special issue on natural experiments is to provide timely evidence to further understand the effectiveness of built environment interventions on health behaviours and chronic disease prevention in a Canadian context. 

*Health Promotion and Chronic Disease Prevention in Canada: Research, Policy and Practice* is seeking relevant topical research articles that present new findings or synthesize/review existing evidence on natural experiments of the built environment (or related policies) that influence health behaviours with implications for chronic disease prevention in Canada. 

Relevant topic areas include, but are not limited to:

- Built environments, including community or neighbourhoods, workplaces, schools, transportation infrastructure, home environments, recreation environments, parks, playgrounds, green spaces, public open spaces, natural environments and seniors’ residences.
- All health-related behaviours, including physical activity, sedentary behaviour, sleep, food consumption, smoking and substance use.
- Chronic diseases and health-related outcomes, including body mass index, fitness, blood pressure, blood lipids, blood sugar, injuries, falls, mental health, stress, depression, anxiety, Alzheimer’s disease, dementia, obesity, metabolic syndrome, cardiovascular disease, cancer, diabetes and lung disease.

International submissions will be considered if they include Canadian data, results (e.g. as part of multi-country studies or global comparisons) and/or evidence-based discussion of implications for community or population health in Canada.

Consult the Journal’s website for information on article types and detailed submission guidelines for authors. Kindly refer to this call for papers in your cover letter.

All manuscripts should be submitted using the Journal’s ScholarOne Manuscripts online system. Pre-submission inquiries and questions about suitability or scope can be directed to HPCDP.Journal-Revue.PSPMC@phac-aspc.gc.ca.

Submission deadline: November 30, 2024
